# Dopamine D2 receptor activation counteracts olfactory dysfunction and related cellular abnormalities in experimental parkinsonism

**DOI:** 10.1016/j.heliyon.2024.e35948

**Published:** 2024-08-08

**Authors:** Daniel Medeiros, Débora Masini, Carina Plewnia, Laura Boi, Martha Rosati, Nicolas Scalbert, Gilberto Fisone

**Affiliations:** Department of Neuroscience, Karolinska Institutet, 171 77 Stockholm, Sweden

## Abstract

Olfactory dysfunction is a common non-motor symptom associated with Parkinson's disease (PD). This condition usually appears before the onset of the cardinal motor symptoms and is still poorly understood. Here, we generated a mouse model of early-stage PD based on partial 6-hydroxydopamine (6-OHDA) lesion of the dorsal striatum to reproduce the olfactory deficit and associated cellular and electrophysiological anomalies observed in patients. Using this model, we investigated the effect of long-term, continuous administration of pramipexole, a dopamine D2/3 selective agonist, on olfactory dysfunction. We found that pramipexole reverted the impairment of odor discrimination displayed by the mouse model in the habituation/dishabituation test. In line with similar observations in PD patients, the mouse model showed an increase of dopamine cells paralleled by augmented levels of the dopamine marker, tyrosine hydroxylase, in the olfactory bulb (OB). These changes, which have been proposed to contribute to olfactory dysfunction, were abolished by oral administration of pramipexole. Local field potential recording in the OB of 6-OHDA lesion mice showed reduced oscillations in the beta frequency range, in comparison to healthy control mice. This abnormality, which is suggestive of defective long range OB transmission, was also counteracted by pramipexole. Altogether these findings indicate that prolonged pharmacological stimulation of dopamine D2-like receptors rescues olfactory discrimination observed in experimental parkinsonism. Moreover, they show that this protective effect is exerted in parallel to a normalization of dopamine neurons and beta band oscillations in the OB, providing information on the potential mechanisms involved in PD-related olfactory dysfunction.

## 1 Introduction

Impaired olfaction is among the most frequent non-motor symptoms observed in Parkinson's disease (PD) [1]. This condition often precedes the motor deficits typically observed in PD patients and is therefore regarded as a major prodromal marker [1,2]. The mechanisms at the basis of impaired odor recognition are still a matter of discussion and may be related to modifications of dopaminergic transmission in the olfactory bulb (OB), which is the first region involved in processing odorant sensory signals.

In the OB, dopaminergic interneurons located in the external part of the glomerular layer (GL), which undergo continuous replacement [3], play an essential role in modulating incoming olfactory information [4]. By releasing dopamine these neurons activate local dopamine D2 receptors (D2R), which exert presynaptic inhibition on glutamatergic olfactory terminals within the GL [[5], [6], [7], [8]].

Clinical studies show that PD patients present an increased number of dopamine neurons in the OB [9,10], an effect predominantly observed in women [11]. This phenomenon is also found in non-human primate and rodent models of PD [[12], [13], [14], [15], [16]] and may be at least in part responsible for impaired olfaction. According to this hypothesis, increased dopaminergic transmission in the OB would depress the excitatory synaptic control exerted by primary olfactory terminals on mitral and tufted cells, which are the major output channels of the OB, ultimately resulting in olfactory deficit [9].

The increased number of dopamine OB neurons in PD patients and animal models may result in modifications of local field potential (LFP) associated with dysregulated neuronal transmission. In the OB, changes of oscillatory patterns have been linked to distinct behaviors and cognitive processes [17] and odor-evoked beta and gamma rhythmic bands have been associated to large-scale and local network activity, respectively [18]. Interestingly, in mice with partial depletion of dopamine neurons in the substantia nigra (SN), impaired olfactory behavior has been associated with a reduction of odor-evoked beta and gamma oscillations [19]. In addition, electrobulbogram analysis performed in patients with PD revealed a power reduction in odor-induced theta, beta, and gamma bands [20].

The impairment of odor recognition associated with PD is refractory to the administration of L-DOPA, which also fails to restore the physiological number of dopamine neurons in the OB [[21], [22], [23]]. However, little is known on the possible effects exerted on hyposmia by other anti-parkinsonian medications, including dopamine receptor agonists. In this study, we examined the effect of the dopamine D2-like receptor agonist, pramipexole, on olfactory discrimination in a 6-hydroxydopamine (6-OHDA) mouse model of early-stage PD. In the same model, we also tested the ability of pramipexole to normalize dopamine cells and the consequent increase of the dopamine marker, tyrosine hydroxylase (TH). Finally, we examined the effect of pramipexole on changes in LFP recorded in the OB of the PD mice.

## 2 Methods

### 2.1 Animals

Mice were housed in groups in a climate-controlled environment set at 22 °C, with ad libitum access to food and water. Behavioral, electrophysiological, and Western blot experiments were conducted on C57BL/6J mice (20–25 g, 2–4 months of age). Immunofluorescence experiments were performed in *Slc6a*^Cre^ (DAT-Cre) knock-in [24] crossed with loxP-flanked tdTomato mice (Jackson Laboratory, strain #007909). Female mice were used in all studies, as they are less susceptible to post-operative mortality compared to male mice [25].

### 2.2 Drugs and experimental groups

6-OHDA hydrochloride (Sigma-Aldrich, St. Louis, MO, USA) was dissolved in 0.9 % sterile saline including 0.02 % of ascorbic acid and locally injected in the striatum. Pramipexole dihydrochloride (MedChemExpress, Monmouth Junction, USA) was administered in the drinking water for four weeks following sham- or 6-OHDA-lesion and prior to behavioral experiments. Drug concentration, based on in-cage assessment of liquid consumption, was set at 0.036 mg/ml, resulting in a daily dose of 3 mg/kg. To ensure accurate drug dosing, the liquid intake and body weight were regularly checked. In addition, the drinking water was replaced every other day. Mice were separated into three experimental groups according to treatment: sham lesion (Control), 6-OHDA lesion (6-OHDA), and 6-OHDA lesion treated with pramipexole (6-OHDA + PPX). A total of 57 mice were used, divided into three experiments: olfactory behavior and electrophysiological recording (Control = 9, 6-OHDA = 8, 6-OHDA + PPX = 8), Western blot quantification (Control = 8, 6-OHDA = 9, 6-OHDA + PPX = 8), and immunofluorescence and cell count (Control = 3, 6-OHDA = 3, 6-OHDA + PPX = 3; with analyses performed in three coronal sections per animal, 27 sections in total).

### 2.3 6-OHDA lesion and electrode implant

Following isoflurane anesthesia, mice were positioned in a stereotaxic frame (Stoelting - Europe) on a heating pad set at 37 °C to maintain normothermia. Prior to surgery, the animals were given a subcutaneous injection of Temgesic (0.1 mg/kg; Apoteket, Stockholm, Sweden) for analgesia. An ophthalmic ointment (Oftagel, Santen; Apoteket, Stockholm, Sweden) was applied to prevent corneal damage. The neurotoxin 6-OHDA hydrochloride (4 μg/μL, free base) was infused (1.25 μL) bilaterally into the dorsal striata. The infusion was performed according to the following coordinates relative to Bregma: anteroposterior (AP) +0.6 mm, mediolateral (ML) ± 2.2 mm, and dorsoventral (DV) −3.2 mm. Equivalent volumes of the vehicle solution were injected into sham-lesion (Control) mice. After a recovery period of three weeks, a stainless-steel, Teflon-coated electrode (Model 791400, A-M Systems, USA) was implanted in the right olfactory bulb of the mice, at coordinates relative to Bregma: AP +4.28 mm, ML -1.0 mm, and DV -1.5 mm. A surface micro-screw was placed in the occipital bone to serve as a reference for the signal. The electrode and micro-screw were fused to a Straight Male PCB Header (4-pin) and secured to the skull using dental cement and acrylic. All mice recovered two weeks before the start of the experiments.

### 2.4 Olfactory analysis

Olfactory performance was evaluated by employing a previously described habituation/dishabituation test with minor modifications [26]. The olfactory test started one day after the four-week oral treatment. The animal was connected to the LFP recording system, placed in a clean homecage for 10 min and then presented with a dry cotton swab for 5 min. Each animal was then exposed to a neutral odor (saline wet cotton swab) followed by a sequence of three unique social odors (urine wet cotton swabs, 20 μL/presentation, freshly prepared). Mice were exposed to each social odor type for three periods of 2 min separated by rest intervals of 1 min. Social odors were produced by pooling urine from five adult mice. The two female odor sets (Female 1 and Female 2) were obtained by pooling the urine from five different female mice (out of a total of ten females). The male odor set (Male) was obtained by pooling the urine from five different males. Animals were exposed to Female 1, Female 2 and Male odors in sequential order. During the test, habituation was determined as the progressive decrease in the time spent exploring a specific odor over three successive presentations, and dishabituation was determined as the increase in the time spent exploring the new odor, reflecting the ability to detect olfactory novelty [27]. The test was video recorded with a USB camera (Logitech Brio webcam, 1080 pixels, 10 frames per second) for offline analysis. Olfactory exploration was determined by measuring the time when the mouse nose touched or was oriented toward the cotton swab within a distance of less than 1 cm. Baseline LFP values were extracted from non-exploring epochs when the animal was immobile and not engaged in any exploratory behavior. Exploratory and basal (non-exploring) behaviors were annotated to each video frame by a researcher unaware of the experimental groups. OBS Studio software (Version 27.0.1) was used to record the behavioral and electrophysiological data on a single screen, and to synchronize the datasets.

### 2.5 Measurement of motor activity

The distance traveled during the olfactory test was measured by tracking the position of individual mice using DeepLabCut (version 2.1). The software was trained to identify relevant body parts (head, body center, and tail base). One hundred frames from a representative video were labeled, and 95 % were automatically selected to train the ResNet-50-based neural network, set on default parameters (500k interactions) [28]. All behavioral video recordings were analyzed by the trained neural network. The tracking data were imported into MATLAB, where the x and y positions of the mouse body parts were averaged to produce a single Cartesian coordinate for the mouse location in each video frame. The distance traveled was calculated by taking the square root of the sum of the squared differences between the x and y coordinates (in pixels) of consecutive video frames. The final values of the distance traveled were converted to meters.

### 2.6 LFP recording

The OB electrical signal was amplified 5000-fold and bandpass filtered within the 1 Hz to 3 kHz range using an ERS100C amplifier (Biopac MP160). Data were recorded at a sample rate of 6250 Hz using AcqKnowledge software (version 4.1, Biopac Systems) and subsequently stored in a hard drive for offline analysis. Two behavioral categories observed during the olfactory test were identified and windowed using the synchronized video from OBS: baseline, when the animal was motionless and non-exploring, and olfactory exploration, when the animal was interacting with the odor cue. The power spectral density of the LFP signal was calculated for each period using the MATLAB standard function pwelch (1 Hz steps and no overlapping). Values were normalized by dividing the power in each frequency bin by the total power observed during baseline or olfactory exploration periods. The LFP power in the theta (7–12 Hz), beta (15–35 Hz) and gamma (35–95 Hz) range was assessed for each animal during baseline (cumulative of 20 s) and olfactory exploration periods. The final analysis included only animals with correct placement of OB electrodes.

### 2.7 Immunohistochemistry

Mice were anesthetized with pentobarbital (1:1 in 0.9 % sterile saline) and perfused with 4 % (wg/vol) paraformaldehyde (PFA, Sigma-Aldrich, Darmstadt, Germany) in phosphate-buffered saline (PBS at pH 7.4). Brains were dissected and post-fixed overnight at 4 °C in PFA (4 %). The brains were then rinsed in PBS, encased in agarose 4 %, and cut into coronal sections (50 μm) with a vibratome (LEICA VT 1000S). The sections were stored in cryoprotectant solution (PBS 0.1M, ethylene glycol, and glycerol) at −20 °C until processing for cell counting or verification of electrode placement. Staining was performed on three selected sections of the OB (bregma distance AP +3.92) per animal. Sections were permeabilized with Tris-buffered saline (TBS)/0.1 % Triton X-100 solution and blocked for 90 min in PBS/0.3 % Triton X-100 and normal goat serum. Sections were then incubated overnight at 4 °C with the primary antibody against tyrosine hydroxylase (TH; 1:1000; #AB152, Merck) and subsequently with secondary antibody 1:500 (Alexa Fluor 488 from Jackson and ImmunoResearch) for 1 h at room temperature. Finally, brain sections were mounted on poly-L-lysine prepared glass slides (Sigma diagnostic, USA) and covered with a media of DABCO (glycerol solution containing 1,4-Diazabicyclo [2.2.2] octane powder).

### 2.8 Imaging and cellular counting

Images of the OB were acquired at 20× by confocal microscopy (Zeiss, Axioplan2 imaging, LSM 510 META, Germany) with the Zen software. Colocalization images of TH-staining in DAT-Cre crossed with tdTomato mice (DAT-Td tomato mice) were taken using the Z-Stack function. Cell counts were performed in three sections of the external GL per animal. Images were processed with ImageJ software (Scion Corporation, USA) using plugins for threshold and automated cell count.

### 2.9 Western blot

Mice were sacrificed by decapitation, the head was immediately immersed in liquid nitrogen for 5 s, and the brains were rapidly removed. Striatum and OB from left and right hemisphere were dissected, sonicated in 1 % sodium dodecyl sulfate (SDS), and boiled for 10 min in sample buffer. Samples were cooled down and stored at −20 °C. Aliquots of 2 × 5 μL of each homogenate were used for protein quantification using a BCA assay kit (Pierce, Rockford, IL, USA). Equal protein amounts (25 μg/sample) were separated by SDS-PAGE and transferred overnight to nitrocellulose membranes (0.45 μm, Thermo Scientific, Germany). After the transfer, the membranes were placed on a shaker and washed at room temperature for 5 min in PBS, and then for 1 h in Odyssey blocking buffer. Next, membranes were incubated in primary antibody (anti-TH 1:1000; #AB152, Merck), anti-beta-actin 1:10000 (#A5316, Sigma- Aldrich) at room temperature for 2 h and afterward washed using PBS-Tween (0.1 %). Detection with fluorescent secondary antibody binding (IR Dye 800CW and 680RD, Li-Cor Bioscience, Lincoln, NE, USA) was performed at room temperature for 90 min. Quantification was performed with a Li-Cor Odyssey infrared fluorescent detection system, and the software Image Studio to quantify signal intensities. Data were calculated as % of control and normalized by the corresponding beta-actin protein. Values from both sides of striatum and OB were averaged for final statistical analysis.

### 2.10 Statistics and group comparisons

The GraphPad Prism 9 software was used for statistical analysis. The normal distribution of all datasets was confirmed by Kolmogorov–Smirnov test. Differences in odor exploration time were analyzed with Brown-Forsythe ANOVA, and Welch post-hoc test. The group comparisons of distance traveled, total olfactory exploration time, dopaminergic OB cell counts, TH immunoreactivity and LFP power were analyzed by one-way ANOVA and Bonferroni post-hoc test.

## 3 Results

### 3.1 Pramipexole rescues olfactory discrimination induced by striatal 6-OHDA lesion

**Fig. 1:**
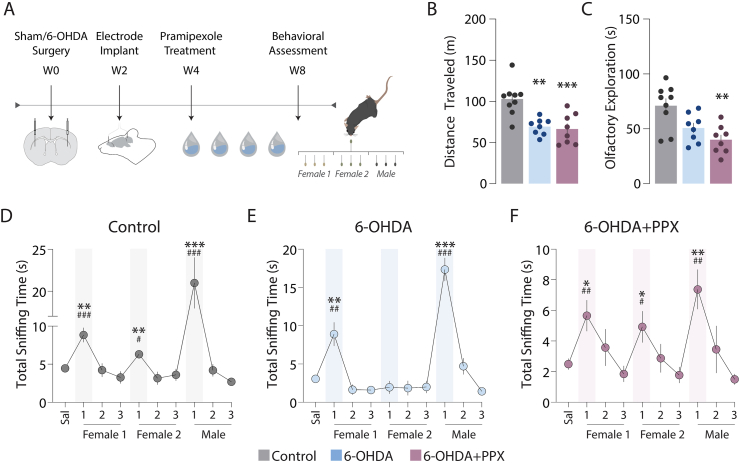
Chronic pramipexole treatment rescues olfactory discrimination in a mouse model of PD. (A) Schematic representation of the experimental design, including the habituation-dishabituation behavioral test performed in mice with a sham lesion (Control), a 6-OHDA lesion (6-OHDA) and a 6-OHDA lesion followed by administration of pramipexole (6-OHDA + PPX). (B, C) Bar graphs showing (B) distance traveled during the olfactory test and (C) total time spent exploring the odors. **p < 0.01 and ***p < 0.001 vs. Control, one-way ANOVA followed by Bonferroni post-hoc test, (B) F_2, 22_ = 12.23 and (C) F_2, 22_ = 7.2. (D–F) Line graphs showing the time (s) spent by Control, 6-OHDA, and 6-OHDA + PPX mice exploring saline or the three social odors from distinct pools of female (Female 1 and Female 2) and male (Male) mice. *p < 0.05, **p < 0.01, ***p < 0.001 vs. dishabituation time, and #p < 0.05, ##p < 0.01, ###p < 0.001 vs. habituation time; Brown-Forsythe ANOVA followed by Welch post-hoc test, F_6, 13_ = 24.1 for (D), F_6, 26_ = 37.3 for (E), and F_6, 27_ = 8.3 for (F).

The ability to discriminate between three different social odors was measured in control, 6-OHDA, and 6-OHDA + PPX mice using the olfactory habituation/dishabituation test (Fig. 1A). A preliminary analysis of motor performance during the test showed a reduction of movement in 6-OHDA and 6-OHDA + PPX mice compared with control mice (Fig. 1B). The 6-OHDA + PPX group also showed reduced odor exploration time in comparison to the control group (Fig. 1C). Because of these differences in motor activity and exploration time the results of the olfactory test are shown separately for each experimental group (Fig. 1D–F). All mice, independently of lesion or treatment, were able to detect the first social odor (Female 1), as shown by the increase in exploration time (Fig. 1D–F). They also displayed habituation to the first social odor, as indicated by the decrease in exploration time between the first and third presentation (Fig. 1D–F). However, in line with previous work [29], 6-OHDA lesion mice did not explore the second (novel) Female 2 odor (Fig. 1E). This deficit in dishabituation, which is indicative of impaired olfactory discrimination, was abolished by chronic administration of pramipexole (Fig. 1F). The presentation of the Male odor induced a large increase in exploration time in all experimental groups (Fig. 1D–F).

### 3.2 Pramipexole normalizes the number of dopamine neurons in the OB of 6-OHDA lesion mice

**Fig. 2:**
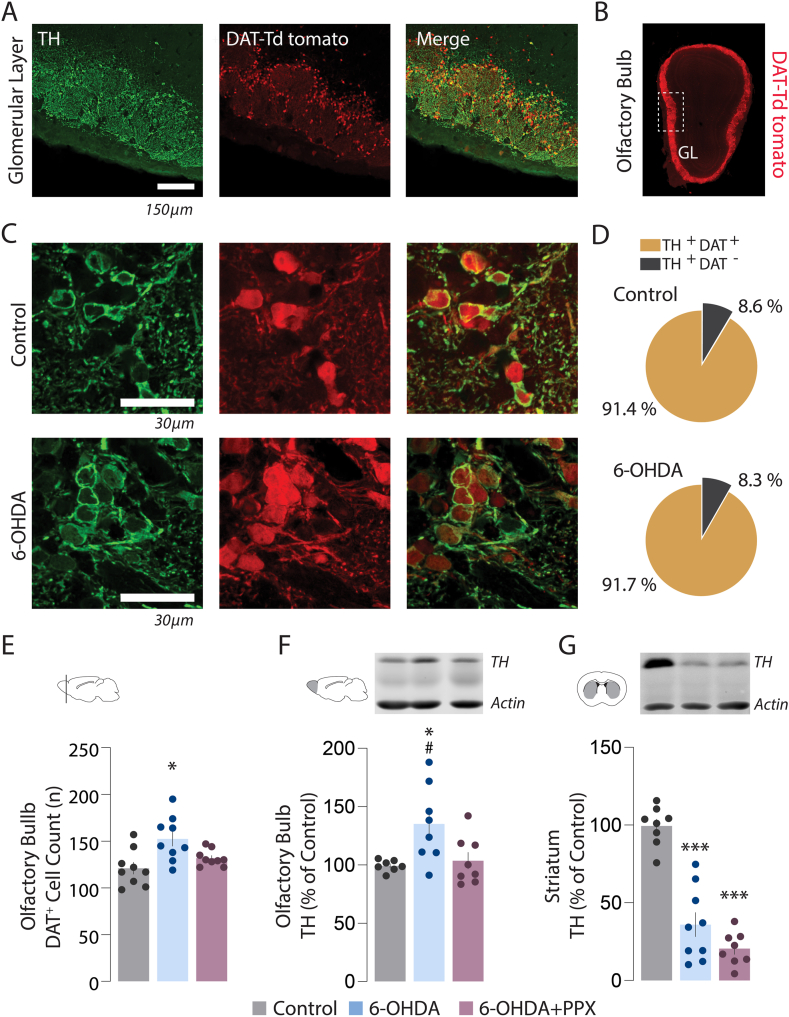
Chronic PPX treatment normalizes the number of TH + cells in the external GL of PD mice (A) Representative images showing co-localization of TH immunoreactivity and tdTomato in GL cells. (B) Distribution pattern of DAT-Td tomato cells in the OB. Note the selective localization in the external GL. (C) Representative immunofluorescence images showing TH and DAT-Td tomato expressing cells in Control and 6-OHDA lesion mice. (D) Pie chart showing the percentage of TH + cells colocalized with the tomato reporter. (E) Number of DAT + cells measured in three individual GL coronal sections/mouse (three mice/group). (F, G) TH-immunoreactivity measured by western blotting in (F) olfactory bulb and (G) striatum. Data are the mean ± SEM. *p < 0.05, ***p < 0.001 vs. Control, and #p < 0.05 vs. 6-OHDA + PPX; one-way ANOVA followed by Bonferroni's post hoc test, F_2,6_ = 11.06 for (E), F_2,20_ = 5.6 for (F), and F_2,22_ = 49.1 for (G). See Supplementary Fig. 1 for uncropped Western blot images.

**Fig. S1:**
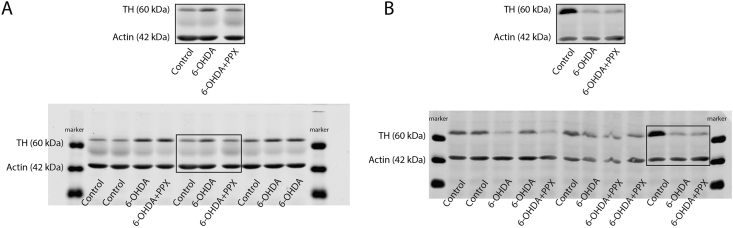
Western blot images showing uncropped membranes of TH and beta-actin immunoreactivity in the olfactory bulb (A) and striatum (B). Representative western blots images shown in Fig. 2F, and G are highlighted.

In a second set of experiments, the number of dopamine cells and the levels of TH, a dopamine marker, were determined in the OB of control, 6-OHDA and 6-OHDA + PPX mice (three coronal sections per mouse). Dopamine cells were counted using a double transgenic mouse line with genetically tagged dopamine transporter expressing cells (DAT-Td tomato mice). Nearly all Td tomato-positive cells were located in the external GL (Fig. 2A and B) and expressed TH (Fig. 2C). Quantification analyses in the medial part of the external GL confirmed that both control and 6-OHDA lesion hemispheres had more than 90 % of TH + cells tagged with the tomato reporter (TH + DAT+) (Fig. 2D). Based on these results, we proceeded by counting the number of DAT + cells in the GL and found that the number of dopamine cells in the 6-OHDA, but not in the 6-OHDA + PPX group, was increased in comparison to the control group (Fig. 2E). These findings were further substantiated by Western blot analysis showing increased TH immunoreactivity in the OB of 6-OHDA mice, in comparison to both control and 6-OHDA + PPX mice (Fig. 2F and Supplementary Fig. 1A). Parallel Western blot analysis of striatal tissue showed a decrease of TH protein of approximately 75 % in 6-OHDA mice, independently of pramipexole administration (Fig. 2G and Supplementary Fig. 1B). Taken together, these results indicate that a partial striatal lesion with 6-OHDA results in increased TH + cells and TH levels within the external GL and that pramipexole fully reverts this effect.

### 3.3 Pramipexole counteracts alterations in OB oscillations caused by 6-OHDA

**Fig. 3:**
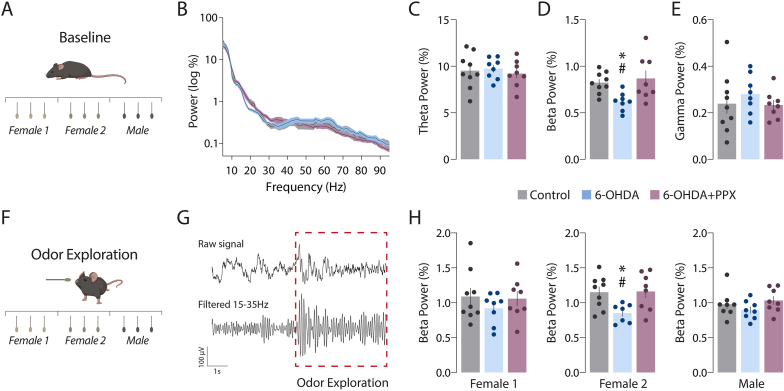
Impaired OB oscillatory activity in PD mice was counteracted by long-term treatment with PPX. (A) Schematic representation of baseline non-exploring period. (B) EEG power spectrum density during baseline. (C–E) LFP power in the OB at theta, beta, and gamma bands. (F) Schematic representation of social odor exposures during the habituation-dishabituation test. (G) Representative raw and beta filtered traces during odor exploration. (H) Combined beta power of the three presentations for each social stimulus. Data are the mean ± SEM. *p < 0.05 vs. Control, and #p < 0.05 vs. 6-OHDA + PPX; one-way ANOVA followed by Bonferroni's post hoc test, F_2,22_ = 0.2 for (C), F_2,22_ = 5.4 for (D), F_2,22_ = 0.5 for (E), and F_2,22_ = 0.7 (Female 1), F_2,21_ = 4.5 (Female 2), F_2,21_ = 1.3 (Male) for (H).

Changes in OB oscillations associated with olfactory dysregulation, and altered neuronal processing were examined during the habituation/dishabituation test. Electrophysiological recordings of baseline OB activity, taken in immobile, non-exploring animals (Fig. 3A and B), showed lower beta power in the 6-OHDA group compared to both control and 6-OHDA + PPX groups (Fig. 3D). No difference was observed in theta or gamma bands (Fig. 3C and E). Based on these findings we proceeded with the analysis of beta oscillations during olfactory exploration (Fig. 3F–H). We found a significant reduction of beta power in 6-OHDA mice during exposure to the Female 2 odor set. This reduction was not observed during the exposure to the Female 1 or the Male odors sets (Fig. 3H).

## 4 Discussion

In this study we show that prolonged treatment with the dopamine D2/D3 agonist, pramipexole, rescues olfactory discrimination in a mouse model of early-stage PD. This effect is exerted in concomitance with the normalization of TH levels and dopamine neurons. We also show that pramipexole corrects for the reduction of beta oscillations observed in the OB of the PD mouse model.

Our results indicate that the deficit in olfactory discrimination observed in the PD model is partial. Indeed, PD mice were still able to distinguish a female odor from other types of social odors, as shown by the large increase in exploration time in response to the presentation of a male odor. In line with these results, it was shown that the present PD model lacks discrimination between non-social odors but retains the ability to differentiate a non-social from a social odor [29].

D2R are abundantly expressed in the OB [30], where they exert presynaptic inhibition on olfactory terminals located in the GL [[5], [6], [7]]. In line with this notion, studies in rats showed that systemic administration or local OB infusion of the D2R agonist, quinpirole, reduces odor discrimination [31,32]. These studies have been obtained in naïve animals and contrast with the present results in the mouse PD model, which show an increased number of dopaminergic OB neurons and a positive effect of pramipexole on olfactory discrimination. In this case, the beneficial effect of the D2R agonist may result from the observed reduction of excess dopamine neurons in the GL and from a subsequent normalization of dopamine transmission in the OB. This possibility is supported by previous work performed in rats, showing that the deficit in olfactory discrimination caused by administration of 6-OHDA in the substantia nigra is counteracted by concomitant injection of the toxin in the OB, which leads to a reduction of dopaminergic neurons [33]. The ability of pramipexole to reduce the number of dopamine neurons in the OB of PD mice may therefore re-establish correct physiological D2R-mediated transmission, which is necessary to preserve odor discrimination [34].

The normalization of the number of dopamine neurons produced by pramipexole in the OB of 6-OHDA lesion mice contrasts with a study performed in rats with a unilateral lesion of the medial forebrain bundle [35]. In this model, repeated oral administration of pramipexole increased the number of newly generated dopamine neurons in the OB. This incongruence may depend on species-specific differences, since prolonged activation of dopamine D2/D3 receptors did not affect neurogenesis in mice [36] and in neural precursor cells derived from human midbrain [37]. Moreover, the present data are based on the measurement of the total number, rather than the number of newly generated TH-positive neurons in the OB.

The long-term treatment with pramipexole implemented in this study is not sufficient to correct for the reduction in motor activity displayed by 6-OHDA lesion mice during the behavioral tests. This may depend on the route of administration - drinking water instead than intraperitoneal or subcutaneous injection - chosen in this study and suggests that a moderate, long-term stimulation of D2R may effectively stabilize the number of dopaminergic neurons in the OB, ultimately improving olfactory performance.

Previous work in mice with a 6-OHDA lesion of the SN showed impaired olfactory performance, without any modification in the number of dopaminergic neurons in the OB [19]. Our finding of increased TH-positive neurons in the OB of 6-OHDA lesion mice, contrasts with this observation and is more in line with a study by Chiu et al. reporting increased dopaminergic neurons in the external GL following 6-OHDA lesion of the SN [12]. It should be noted that, in contrast to most previous studies [12,19,38,39], the present experiments were performed in female mice, which, aside from displaying a lower post-surgical mortality, may also represent a better model to reproduce the cellular changes described in parkinsonian patients. Indeed, Huisman and colleagues [11], but see also [10], showed that the number of dopaminergic neurons in the OB of healthy control males is approximately two-fold of that found in females, and that these cells are increased in female, but not in male, parkinsonian patients. These observations point to sex differences in the modulation exerted by the dopaminergic system on olfactory function and raise the possibility that in PD patients the efficacy of therapeutic interventions on odor discrimination may also vary according to the gender.

LFP recording during the olfactory discrimination test revealed a general reduction of baseline beta power, but not of gamma or theta power, in 6-OHDA lesion mice compared to control group. In PD mice, decreased beta power was also observed during the exploration of the second female social odor. This finding is in line with previous evidence of decreased odor-evoked beta power in the OB of a mouse model of PD and in parkinsonian patients [19,20]. Long-term administration of pramipexole to 6-OHDA lesion mice resulted in a general recovery of beta power to control level. Notably, in PD mice, the ability of pramipexole to re-establish enhanced beta power during the exploration of the second female odor coincided with the recovery of odor discrimination. A similar correlation between increased beta power and olfactory dishabituation was also observed when PD mice were exposed to the male social odor. This suggests that the changes in the dopamine system generated by this model of PD exert a prominent impact on long-range OB transmission, which has been proposed to correlate with beta oscillatory activity [17].

In this study we characterized a mouse model of PD-related olfactory impairment which recapitulates parallel cellular, and electrophysiological abnormalities associated with this disturbance. Using this model, we provide novel findings indicating that continuous pharmacological activation of D2R counteracts olfactory dysfunction and that this effect occurs in parallel to the normalization of dopamine cells, TH levels and beta band power in the OB.

## 5 Limitations of the study

Although extensively used to study PD symptomatology, 6-OHDA induces a rapid neurodegeneration of dopamine neurons which does not reproduce the progressive nature of the disease. Furthermore, 6-OHDA lesion mice present no Lewy body inclusions, a pathological hallmark of PD which might be involved in olfactory dysfunction. The counting of dopaminergic neurons in the OB was based on a limited number of mice (three/group), which may represent a bias in the analysis. This is partially corrected by the parallel quantification of TH (a marker of dopamine neurons) in the OB. Sham-lesion (control) mice treated with PPX were not included in this study. Although injection of a similar drug (i.e., quinpirole) in the OB of naïve rats impairs olfactory discrimination, the exclusion of this experimental group precludes the possibility to determine unequivocally whether the effect of PPX occurs specifically in the PD model.

## Ethics statement

The research was carried out in compliance with the guidelines established by the Research Ethics Committee of Karolinska Institutet, Swedish Animal Welfare Agency (ethical permit 12148-17), and European Community Council Directive 86/609/EEC.

## Data availability statement

Any additional information about this work and all raw data will be made available on request to Gilberto Fisone (gilberto.fisone@ki.se) or Daniel Medeiros (daniel.medeiros@ki.se). The code used to analyze oscillations in the OB is available at: https://github.com/dacamemg/Olfactory-Bulb-Oscillations.

## CRediT authorship contribution statement

**Daniel Medeiros:** Writing – original draft, Investigation, Formal analysis, Conceptualization. **Débora Masini:** Writing – original draft, Methodology, Formal analysis, Conceptualization. **Carina Plewnia:** Investigation, Formal analysis. **Laura Boi:** Investigation, Formal analysis. **Martha Rosati:** Investigation, Formal analysis. **Nicolas Scalbert:** Investigation. **Gilberto Fisone:** Writing – original draft, Supervision, Methodology, Funding acquisition, Conceptualization.

## Declaration of competing interest

The authors declare that they have no known competing financial interests or personal relationships that could have appeared to influence the work reported in this paper.
